# The Role of Mitochondrial Dysfunction in CKD-Related Vascular Calcification: From Mechanisms to Therapeutics

**DOI:** 10.1016/j.ekir.2024.05.005

**Published:** 2024-05-14

**Authors:** Junmin Huang, Junfeng Hao, Peng Wang, Yongzhi Xu

**Affiliations:** 1 Guangdong Provincial Key Laboratory of Autophagy and Major Chronic Non-communicable Diseases, Key Laboratory of Prevention and Management of Chronic Kidney Disease of Zhanjiang City, Institute of Nephrology, Affiliated Hospital of Guangdong Medical University, Zhanjiang, Guangdong, China

**Keywords:** CKD, mitochondria, osteogenic transdifferentiation, vascular calcification, vascular smooth muscle cell

## Abstract

Vascular calcification (VC) is a common complication of chronic kidney disease (CKD) and is closely associated with cardiovascular events. The transdifferentiation of vascular smooth muscles (VSMCs) into an osteogenic phenotype is hypothesized to be the primary cause underlying VC. However, there is currently no effective clinical treatment for VC. Growing evidence suggests that mitochondrial dysfunction accelerates the osteogenic differentiation of VSMCs and VC via multiple mechanisms. Therefore, elucidating the relationship between the osteogenic differentiation of VSMCs and mitochondrial dysfunction may assist in improving VC-related adverse clinical outcomes in patients with CKD. This review aimed to summarize the role of mitochondrial biogenesis, mitochondrial dynamics, mitophagy, and metabolic reprogramming, as well as mitochondria-associated oxidative stress (OS) and senescence in VC in patients with CKD to offer valuable insights into the clinical treatment of VC.

VC is associated with aging and various diseases and is especially prevalent in patients with diabetic nephropathy and CKD. Following the development of severe VC, vascular stiffness increases, and compliance concomitantly decreases, leading to a 3- to 4-fold increased risk of death and cardiovascular disease,^1^ which is the primary cause of mortality in patients with end-stage renal disease.^2^ In the context of end-stage renal disease, the pathological deposition of calcium salts in the arterial wall becomes extremely active. In addition, coronary artery calcification was observed to be independently and significantly correlated with the risk of myocardial infarction and heart failure in patients with CKD.^3^ Moreover, arterial calcification severely affects the long-term patency rate of arteriovenous fistulae.^4^ There is currently no unified consensus on how to delay or halt the calcification process in patients with CKD.

CKD-related calcification is hallmarked by medial calcification. VC is a long-term and controllable active pathological process.^5^ The recognized mechanisms underlying calcification involve calcium and phosphorus disorders, lower levels of calcification inhibitors, osteogenic differentiation of VSMCs, cell apoptosis, and reorganization of vascular extracellular matrix. Unlike other smooth muscle cells, VSMCs do not undergo terminal differentiation and exhibit significant plasticity.^6^ Their osteogenic transformation serves as the pathophysiological basis of VC in CKD. Under sustained stimuli such as mineral disorders and uremic toxins, the expression of contractile markers is down-regulated in VSMCs, whereas that of osteogenic markers, including bone morphogenetic protein 2 (BMP2) and runt-related transcription factor 2 (RUNX2), is upregulated.^7^^,^^8^ The transcriptional activation of osteogenic genes in vascular cells plays a crucial role in VC in CKD.

Mitochondria are key cellular energy factories that synthesize adenosine triphosphate (ATP) and various metabolic substrates in the oxidative respiratory chain. Reactive oxygen species (ROS) is a by-product of the respiratory activity of the electron transport chain and plays an instrumental role in different aspects as a key signaling molecule.^9^, ^10^, ^11^ Mitochondrial homeostasis is comaintained by mitochondrial biogenesis, fission, and fusion, and mitophagy. As energy-intensive cells, VSMCs are susceptible to the influence of mitochondrial morphology and dysfunction. It is worthwhile noting that mitochondria are not only an important source but also a target of uremic toxins.^12^ The accumulation of uremic toxins, including proinflammatory cytokines and ROS, stimulates the osteogenic differentiation of VSMCs through different downstream pathways, with mitochondrial dysfunction serving as a central mediator.^13^

Although the exact mechanism of VC in CKD remains elusive, mounting evidence indicates that mitochondrial dysfunction and metabolic reprogramming are key factors that are implicated in VC in CKD and induce the transformation of VSMCs into an osteogenic phenotype.^14^, ^15^, ^16^ To identify the significance of mitochondrial dysfunction in VSMCs osteogenic differentiation in VC in CKD, this article aimed to outline recent advancements in related fields and explore potential clinical treatments for improving VC outcomes in patients with CKD.

### Mitochondrial Biogenesis in VC

As a dynamic organelle, mitochondrial biogenesis is a regenerative program that maintains mitochondrial numbers, replacing old and damaged mitochondria with new and healthy ones to meet cellular energy needs. This process is largely dependent on the regulation of peroxisome proliferator-activated receptor gamma coactivator-1α (PGC-1α). PGC-1α predominantly activates 2 key nuclear transcription factors, namely nuclear factor E2-related factor 1 and nuclear factor E2-related factor 2, which in turn trigger the transcription of the oxidative phosphorylation system protein encoded by mitochondrial transcription factor A and nuclear DNA.^17^ Meanwhile, mitochondrial transcription factor A is responsible for governing the transcription and replication of mitochondrial DNA, with the oxidative phosphorylation system protein being an integral component of the oxidative respiratory chain. Multiple pathways such as cGMP, AMP-activated protein kinase (AMPK), and sirtuin (SIRT) 1 have been established to regulate the expression of PGC-1α.^18^ Previous studies demonstrated that under high phosphate-simulated uremia conditions, the former collaborates with laminar shear forces to induce mitochondrial biogenesis, initiating the osteogenic transformation of VSMCs through the integrin β1-ERK1/2 signaling pathway, as evidenced by the upregulation of mitochondrial transcription factor A and mitochondrial DNA polymerase γ.^14^ However, inhibition of PGC-1α expression weakens the maximum respiratory capacity of mitochondria, thereby enhancing the production of superoxide and mitochondrial ROS. In turn, this promotes the expression of RUNX2 and sex determining region Y-box 9 and the osteogenic transdifferentiation of VSMCs, ultimately driving VC.^16^ The rationale behind this phenomenon may be ascribed to increased energy demands during the phenotype transformation process of VSMCs, inducing an initial increase in compensatory mitochondrial biogenesis.^19^ For example, PGC-1α expression is upregulated in VSMCs and activates mitochondrial biogenesis as a response to early hypoxic damage.^20^

Indeed, restoration of mitochondrial biogenesis is effective against VC. According to a previous study, metformin promotes PGC-1α expression and downregulates the expression of 2 osteogenic gene markers (RUNX2 and BMP2) in β-glycerophosphate (β-GP)-treated VSMCs in a dose-dependent manner. Mechanistically, metformin interferes with the activation of β-GP-induced apoptotic gene pyruvate dehydrogenase kinase 4 (PDK4) through the AMPK-PGC-1α signaling pathway.^21^ In VSMCs, PDK4 expression was found to be upregulated in the Pi-induced calcification model. As a downstream factor, SIRT3 mediates the effects of PGC-1α in inhibiting mitochondrial ROS production and calcium deposition by deacetylating superoxide dismutase 2, a major antioxidant in mitochondria.^16^^,^^22^ In the future, the PGC-1α/ SIRT3 pathway may be a target for optimizing the osteogenic transformation of VSMCs. In addition, resveratrol supplementation has been shown to attenuate VC, possibly mediated by upregulation of mitochondrial biogenesis via the AMPK or SIRT 1/Nrf2 signaling pathway.^23^ Overall, the aforementioned *in vivo* and *in vitro* experiments established that PGC-1α overexpression can inhibit osteogenic transformation in VSMCs and subsequently attenuate VC, indicating that it is a powerful endogenous protective agent against calcification (Figure 1).

**Figure 1:**
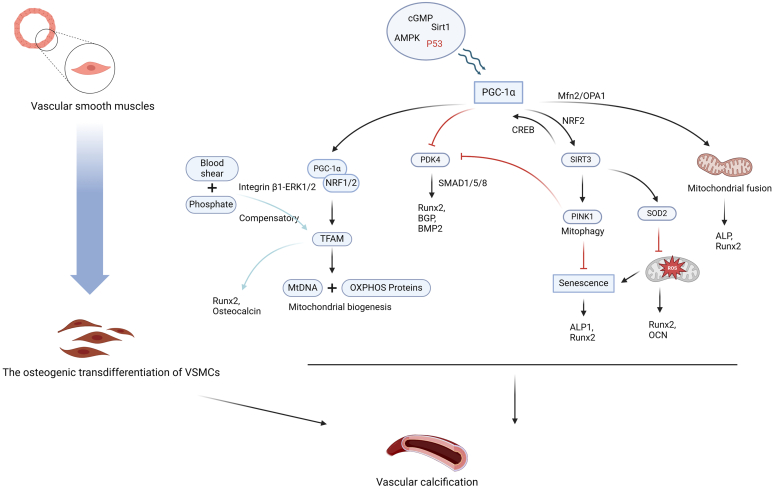
Mitochondrial biogenesis with PGC-1α as the regulatory center is highly involved in the osteogenic transdifferentiation of VSMCs through apoptosis, mitochondrial dynamics, cell autophagy, etc., (Created with BioRender.com). PGC-1α, peroxisome proliferator-activated receptor gamma coactivator-1α; VSMCs, vascular smooth muscles.

### Mitochondrial Dynamics in VC

Mitochondrial dynamics include mitochondrial division and fusion, and their balance plays a vital role in the role and form of mitochondria. They participate in meeting cellular-specific energy demands and responding to external signals.^24^ In mammalian cells, mitochondrial fusion is coregulated by outer mitochondrial membrane proteins and inner mitochondrial membrane proteins, the former comprising mitofusin 1 and mitofusin 2; whereas the latter, optic nerve atrophy factor 1, catalyzes mitochondrial inner fusion.^25^

Fission is largely carried out by dynamin-related protein 1 (DRP1) and fission protein 1, with calcineurin dephosphorylates DRP1 recruiting it to the mitochondrial surface to interact with mitochondrial fission factor, thereby promoting fission.^26^ As is well documented, mitochondrial fission is the foundation of early cell apoptosis. Pore-forming protein and DRP1 are colocated at the fission site and are implicated in ridge remodeling.^27^ The cytochrome c in mitochondria is redistributed from the cristae to the membrane gap and released into the cytoplasm, triggering the sequential activation of the caspase-9 and -3 cascades.^28^ Pathological stimuli elicit excessive mitochondrial division in VSMCs, generating a large amount of mitochondrial debris and ROS that eventually facilitate apoptosis.^29^ Lipoic acid prevents OS-induced VC by inhibiting VSMC apoptosis and restoring the Gas6/Axl/Akt survival pathway.^30^

Mitochondrial dynamics disorders are intricately linked to VC. DRP1 is enriched in calcified regions of human carotid arteries and has been found to exacerbate VC in an OS-dependent manner.^31^ A previous study determined that p53 aids in transferring DRP1 from the cytoplasm to mitochondria to promote fission and effectively inhibit the expression of mitophagy protein, BCL2-interacting protein 3, which may contribute to the osteogenic transformation of VSMCs and ultimately VC.^32^^,^^33^ Lactic acid upregulates DRP1-mediated mitochondrial fission and suppresses BCL2-interacting protein 3-induced mitophagy through the DNA-PKca/p53 pathway by activating nuclear receptor 4A1, significantly increasing the transcription of RUNX2 and BMP2 proteins in calcified VSMCs.^34^ Eliminating damaged mitochondria through a fission-dependent autophagy pathway is manifested by AMPK phosphorylating the DRP1 receptor mitochondrial fission factor to support mitochondrial autophagy.^35^ In the absence of sufficient energy, AMPK activates DRP1 phosphorylation and translocation to induce mitochondrial division and maximize ATP production.^36^ However, other studies have concluded that AMPK essentially serves as an upstream negative regulator for DRP1. AMPK reduces DRP1-mediated mitochondrial division, ultimately down-regulating the expression level of RUNX2 in VSMCs. Inhibition of AMPK phosphorylation mitigates the protective effect against VC.^15^^,^^37^

The effect of mitochondrial fusion on VC contrasts with that of DRP1-driven mitochondrial fission; that is, the activation of optic nerve atrophy factor 1 can delay the occurrence of calcification. Melatonin promotes mitochondrial fusion through the AMPK/optic nerve atrophy factor 1pathway, significantly reducing phosphate-induced calcium deposition, as well as the expression of alkaline phosphatase and RUNX2. Conversely, the removal of optic nerve atrophy factor 1 attenuates the protective effect of melatonin against VC.^38^ The mechanism underlying the effects of mitochondrial division and fusion on the osteogenic phenotype of VSMCs warrants further investigation. (Figure 2).

**Figure 2:**
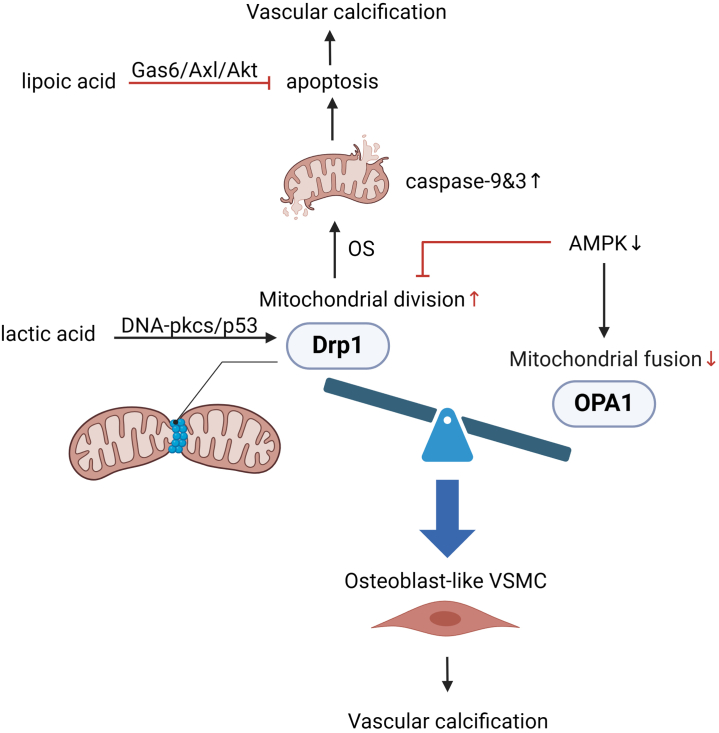
DRP1-driven mitochondrial division promotes the osteogenic transdifferentiation of VSMCs and is regulated by melatonin, lactic acid, etc., whereas mitochondrial fusion exerts a protective role against VC (Created with BioRender.com). DRP1, dynamin-related protein 1; VC, vascular calcification; VSMCs, vascular smooth muscles.

### Mitophagy in VC

Mitophagy is a mechanism that plays a key role in maintaining mitochondrial mass. When damaged mitochondria cannot be cleared by autophagy, the resulting ROS storm eventually leads to programmed cell death. Mitophagy is a type of selective autophagy that reuses amino acids and fatty acids through successive steps.^39^ At present, its mechanisms can be divided into 2 categories, namely the ubiquitin-dependent pathways and nonubiquitin-dependent pathways. The ubiquitin-dependent pathway that has been extensively explored is the PTEN-induced putative kinase 1/Parkin pathway. Following mitochondrial damage, the membrane potential decreases, and PTEN-induced putative kinase 1, which was originally transferred to inner mitochondrial membrane and rapidly decomposed, stably accumulates on the outer mitochondrial membrane, thereby recruiting and activating Parkin. Moreover, mitofusin 2 is phosphorylated by PTEN-induced putative kinase 1 and acts as the receptor for Parkin in mitochondria.^40^ Afterward, the activated Parkin ubiquitinates proteins on mitochondria, facilitating the recruitment of p62 (a bridging protein that binds ubiquitin to LC3). This interaction leads to the clearance of damaged mitochondria by autophagy, occurring at the intersection of mitochondria and the endoplasmic reticulum.^41^ There are numerous LC3-interacting region-containing proteins on the outer mitochondrial membrane, including the NIX and BCL2-interacting protein 3 receptors that act as autophagy receptors to directly bind to LC3 without ubiquitination to initiate mitophagy.^42^^,^^43^

Notably, mitophagy plays a central role in VC by protecting VSMCs from OS and senescence. CKD progression results in impaired gluconeogenic function, accompanied by a decrease in glucose production and lactate clearance.^44^ As the product of glycolysis, lactate has been reported to assist in expediting the osteogenic transformation of VSMCs and VC by disrupting mitochondrial mass^34^ with poly (ADP-ribose) polymerase 1/DNA polymerase γ signaling mediating the above-mentioned effects, which in turn leads to the compensatory upregulation of mitochondrial uncoupling protein 2 and inhibition of mitophagy. Knockout of uncoupling protein 2 in VSMCs down-regulates the mRNA expression of lactate-induced RUNX2 and BMP2 and represses DRP1-mediated mitochondrial fission in VSMCs, ultimately alleviating calcification.^45^ Importantly, mitochondrial fission is closely related to mitophagy. Following a reduction in mitochondrial membrane potential, mitophagy is triggered by promoting mitochondrial separation and early contact between mitochondria and lysosomes.^46^ During mitophagy, Parkin simultaneously ubiquitinates PARIS and promotes the activity of PGC-1, a transcription factor, resulting in crosstalk with mitochondrial biogenesis.^47^ Furthermore, the expression of BCL2-interacting protein 3 is inhibited by the lactate/nuclear receptor 4A1/DNA-PKca/p53 axis, attenuating mitophagy and subsequently accelerating the progression of VC.^48^ Distinct from the dual effects of autophagy, the activation of mitophagy in existent cells protects against VC, and the increase in mitophagy flux attenuates apoptosis. In order to delay the onset of VC, further evidence is necessitated to identify upstream factors that modulate VSMC mitophagy^49^ (Figure 3).

**Figure 3:**
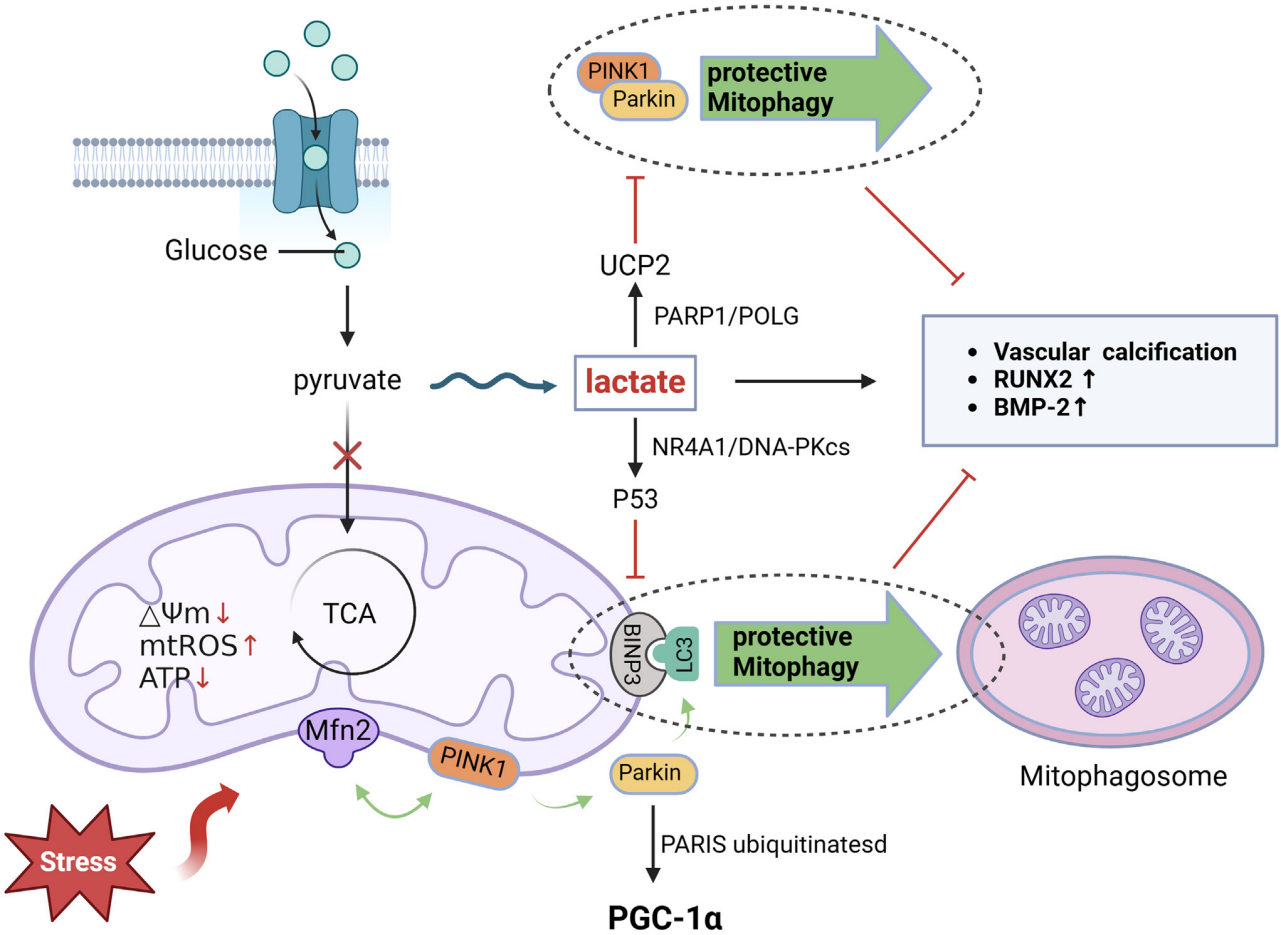
Mitophagy and mitochondrial biogenesis preserve mitochondrial mass and protect against cellular damage. Lactic acid exerts a strong promoting effect on the osteogenic differentiation of VSMCs by interfering with mitophagy, including both ubiquitin-dependent and non-ubiquitin-dependent pathways (Created with BioRender.com). VSMCs, vascular smooth muscles.

### Metabolic Reprogramming in VC

It is widely recognized that even under physiological conditions, VSMCs exhibit high levels of aerobic glycolysis and lactate production, similar to the Warburg effect in cancer cells.^7^ Glycolysis generates ATP by converting glucose to pyruvate, and approximately 45% of the ATP supply in VSMCs is derived from aerobic glycolysis.^50^ However, the preference of VSMCs for the less ATP-efficient metabolic mode of glycolysis remains unclear. It was previously thought that VSMCs may respond to greater energy demands by relying on rapid ATP supply, such as glycolytic reserve capacity.^51^ Nonetheless, a growing number of studies suggest that mitochondrial glycolysis is involved in regulating phenotypic transitions in VSMCs, particularly during VC.^52^

Glycolytic flux is tightly controlled by PFKFB. Noteworthily, the knockdown of PFKFB3 significantly inhibited the expression of osteoblast markers such as alkaline phosphatase l and osteocalcin in VSMCs. PFKFB3-mediated glycolysis promotes the osteogenic transdifferentiation of VSMCs by regulating FoxO3 expression and lactate production.^53^ Periosteal proteins exert their effects by inhibiting mitochondrial oxidative phosphorylation and hyperactivating glycolysis, as well as promoting β-catenin expression and down-regulating PPARc protein levels *in vivo* and *in vitro* to contribute to the development of VC. When blocked with hexokinase inhibitors, the calcification of VSMCs was significantly inhibited.^54^ Furthermore, the Wnt/β-catenin signaling pathway simultaneously mediates the effect of osteocalcin in the pathology of arterial calcification, contingent upon a reduction in maximal mitochondrial respiratory capacity, including a significant decrease in glycolytic capacity and reserve.^55^ As a promoter of glycolysis, PDK4 promotes the osteogenic transdifferentiation of VSMCs by phosphorylating SMAD1/5/8 and enhancing BMP2 signaling.^56^ Conversely, PDK4 promotes mitochondrial fragmentation, impairs mitochondrial respiratory capacity, and drives the metabolic reprogramming of VSMCs toward the Warburg effect. Interestingly, inhibition of glycolysis by 2-DG accelerates calcium deposition in VSMCs despite reducing β-GP-induced lactate production.^57^ As can be deduced, enhanced glycolysis may be an adaptive or protective mechanism for the calcification of VSMCs against energy depletion or OS induced by pathogenic factors. Other studies also concluded that reduced mitochondrial phosphorylation precedes a declined glycolytic capacity under pathological conditions.^55^

In CKD, hyperphosphatemia is an integral pathological factor promoting VC.^58^ Unexpectedly, the rates of glycolysis and ATP production are not altered in VSMCs exposed to β-GP. Instead, VSMCs have a higher oxidative phenotype, suggesting an increased respiratory reserve capacity.^59^ Discrepancies between studies may be ascribed to the cell type and the medium used; osteogenic differentiation of VSMCs induced by osteogenic and calcium phosphate media is mediated by different mechanisms, considering the substantial differences in the transcriptomic datasets regulating calcification genes and inconsistencies in mitochondrial energy metabolism alterations.^51^ In addition, even identical smooth muscle cells from different vascular beds exhibit different calcification tendencies.^60^ A study proposed similar conclusions, that is, oxidative phosphorylation system expression is upregulated in high phosphate-treated VSMCs, but the rates of glycolysis and lactate production remain unchanged.^61^

Although perspectives diverge, (i) glucose has been recognized as a major energy substrate for VSMCs, with VSMCs exhibiting enhanced glucose uptake and metabolism upon phenotypic transition; (ii) aberrant mitochondrial metabolic signaling is involved in the progression of calcification, whereas upregulation of mitochondrial oxidative phosphorylation system in CKD promotes the osteogenic transdifferentiation of VSMCs. Notwithstanding, the following remains to be elucidated: (i) the effect of glycolysis in VC across various disease backgrounds and (ii) the underlying mechanism by which phosphate causes mitochondrial dysfunction in VSMCs. In addition, it is important to acknowledge that endothelial cells and extracellular matrix play an important role in the osteogenic differentiation of VSMCs.^62^^,^^63^ It is challenging to account for all interactions in *in vitro* cell culture models, necessitating further validation through *in vivo* studies.

### OS in VC

Oxidative phosphorylation of mitochondria and glycolysis are the 2 major intracellular ATP-generating pathways. Oxidative phosphorylation is mostly accomplished through the mitochondrial-electron transport chain,^64^ which generates approximately 90% of intracellular ROS.^65^ β-GP inhibits mitochondrial respiration and promotes OS by inhibiting complex I and complex III of the oxidative respiratory chain in VSMCs,^66^ whereas complex I is the primary source of mitochondrial ROS, ascribed to a reduction in mitochondrial proton and electron leakage and coupling efficiency.^67^

ROS accumulation can occur during the early stage of CKD and aggravates renal damage.^68^ Patients with advanced CKD experience an excessive ROS status due to excessive endotoxin, loss of antioxidants during dialysis, and complement and inflammatory cell activation.^69^ OS is caused by an increase in the production of ROS and plays a central role in the pathological mechanism underlying CKD-related VC.^70^^,^^71^ In particular, the uremic environment triggers vascular OS, which is a key event leading to catastrophic mineral ion influx into the VSMC layer and is closely related to the osteogenic reprogramming of VSMCs.^72^

In CKD, calcium phosphate is eventually converted to insoluble hydroxyapatite, which acts as the cornerstone of calcified vessels and stimulates the osteogenic transformation of VSMCs in a concentration-dependent manner. During this process, the decrease in mitochondrial membrane potential and the concurrent increase in intracellular ROS levels, coupled with the normal membrane potential, is a prerequisite for oxidative phosphorylation, establishing a positive feedback loop that promotes OS.^73^ Mitochondrial membrane depolarization, subsequent Ca^2+^ overload, and ERK1/2 activation pathway may mediate the excessive *Pi*-induced OS-related osteogenic differentiation of VSMCs.^74^ Studies have consistently documented that high *Pi* increases *Pi*C abundance through the ERK1/2-mTOR signaling pathway and that silencing mitochondrial phosphate transport genes can relieve the high *Pi*-induced reduction in ROS production and calcification.^75^ *Pi*C is the primary route for *Pi* entry into mitochondria, which is simultaneously associated with ATP generation and the opening of the PT pore. Therefore, its pathophysiological role in VC requires further exploration.^76^ When supplemented with NAC and mitochondrial complex I inhibitors, a reduction in mitochondrial ROS levels and a reversal of hypoxia-induced upregulation of osteogenic genes RUNX2, SOX9, and osteocalcin were observed.^77^ OS damage is the core issue caused by mitochondrial dysfunction, and existing evidence suggests that mitochondrial antioxidants are a promising option for the treatment of VC. However, targeted antioxidants have not yielded satisfactory results in translational medicine. Some scholars postulate that different organelles have inconsistent redox statuses and undergo dynamic changes, highlighting the need for precise antioxidant therapies.^78^ At the same time, there is evidence indicating that mitochondrial-derived peroxides cannot directly damage the DNA of chromatin. Nonetheless, targeting mitochondrial oxidation to VC holds promise for future applications.

### Cellular Senescence During VC

Cellular senescence is a state of irreversible cell cycle arrest, accompanied by an increased expression of aging-related proteins, such as P53, P21, P16, and β-Gal. It is stimulated by various stress conditions, including elevated ROS levels, inflammatory factors, calcium and phosphorus metabolic disorders, and hyperglycemia.^79^ A persistent inflammatory microenvironment and premature senescence are biomarkers of the uremic phenotype. The specific secretory phenotype of senescent cells contributes to the physiological and pathological consequences in organisms, possibly by expelling chemical mediators, thereby further spreading OS and inflammatory phenotypes to adjacent cells.^80^ Damaged organelles and accumulation of abnormal proteins during cellular aging affect mitochondria, and in turn, mitochondrial dysfunction promotes cellular senescence.^10^ Mitochondrial dysfunction has been linked to the senescence of VSMCs. Indeed, senescent VSMCs tend to acquire an osteogenic phenotype, as evidenced by significant upregulation in alkaline phosphatase and RUNX2 expression.^81^, ^82^, ^83^

Circulating Klotho levels are often decreased in CKD, and Klotho deficiency is a model of premature aging. Klotho has been shown to maintain VSMC phenotype by regulating mitochondrial metabolic patterns.^84^ It is critical to further investigate the protective effect of Klotho on mitochondria against VC. In addition, in premature aging mouse models, the reduced accumulation of pyrophosphate due to aberrant mitochondrial function and reduced ATP utilization in VSMC cells eventually leads to excessive VC.^85^ Senescence may act as a mediator of CKD and is an important risk factor for CKD-related VC. The expression of the senescence marker P53 is upregulated in calcified VSMCs. Besides, previous studies have demonstrated that P53 promotes mitochondrial elongation and cellular senescence through DRP1 phosphorylation.^33^ Telomere loss activates P53 and inhibits PGC-1α, ultimately affecting mitochondrial biogenesis and respiratory function, thereby linking mitochondrial function to cellular senescence.^86^

Several studies support the theory that VSMC senescence leads to vascular lesions such as calcification, with VC serving as a marker of vascular stiffness and senescence.^23^ Exosomes secreted by endothelial cells promote VSMC senescence and osteogenic transdifferentiation by inducing a decrease in VSMC mitochondrial membrane potential and functional protein expression.^87^ Recent research indicates that GATA binding protein 6 may be an important regulator in VSMC senescence and osteogenic differentiation. GATA binding protein 6 is upregulated in the calcified aorta of mice and accelerates the osteogenic transdifferentiation of VSMCs by interfering with DNA repair. More importantly, SIRT6 represses GATA binding protein 6 transcription through deacetylation and then protects mice against arterial senescence and calcification.^88^ The specific mechanisms by which CKD-related aging leads to VC remain enigmatic.

### Mitochondria-Targeted Therapy

Although VSMC differentiation represents a response to injury-induced changes, nonreversible VC may occur even during the early stages of kidney disease.^89^ VSMC transdifferentiation is a pivotal step in VC. Currently, there are no effective therapies that specifically target the regression of vascular or valvular calcification. In addition, an increasing number of studies are limited to animal or cellular experiments, and there is a paucity of convincing, controlled, and relevant clinical studies.^90^ Given the compromised metabolic ability of patients with CKD, nonnegligible biological toxicity and dialysis compatibility of drugs should be considered. Although preliminary results from the ongoing clinical cohort studies of VC in patients with CKD are not satisfactory, treatment with sodium thiosulfate appears to be the most promising.^91^ A meta-analysis further shows the effectiveness of sodium thiosulfate in delaying VC and atherosclerosis in hemodialysis patients.^92^ Recently, in order to more effectively inhibit osteogenic phenotype transformation of VSMCs, a biomimetic nanocarrier has been developed to precisely deliver sodium thiosulfate to VSMCs.^93^ At present, the protective effect of sodium thiosulfate on brain cells in CKD model is proved to be related to improvement of mitochondrial function.^94^ However, the therapeutic effect of sodium thiosulfate on VSMCs is still unclear. Therefore, it is necessary to further verify whether the protective effect of sodium thiosulfate on VC is achieved by targeting mitochondrial function.

There is limited relevant research exploring the role of mitochondrial function on VC in patients with CKD. Nevertheless, *in vivo,* and *in vitro* findings support the idea that therapeutic approaches to maintain mitochondrial homeostasis may be beneficial in ameliorating VC in the mouse CKD model. Recently, restoration of miR-30b was observed to attenuate aortic calcification in 5 of 6 nephrectomized rats, which may be mediated by down-regulation of SOX9 expression, preservation of mitochondrial MMP, and enhanced mitophagy pathway activation.^95^ In addition, novel insights into the pathology of uremia-induced VC have been reported. Carbamylation of ATP synthase subunits α and β in mitochondria leads to MMP alterations and dysfunction, inducing OS and inhibiting the expression of ectonucleotide pyrophosphate/phosphodiesterase 1 in VSMCs, and ultimately aggravating arterial calcification. These effects were alleviated following the administration of mitochondrial oxidants.^96^ Not only antimitochondrial oxidators but also interventions targeting mitochondrial biogenesis, inhibition of mitochondrial fission, restoration of mitophagy, and modulation of mitochondrial DNA effectively relieved VC in CKD models. Consequently, the relevant potential interventions were listed in this study. Despite our limited understanding of alterations in mitochondrial function in CKD-related VC, given that mitochondrial dysfunction is a common causative factor, the context listed in this table was not limited to CKD^97^, ^98^, ^99^, ^100^, ^101^ (Table 1).

**Table 1:** Potential interventions targeting mitochondrial function for the treatment of calcification

Agents	Cellular	Animals	Mitochondrial function-related						Signaling Pathway	Target molecule	Osteogenic gene- phenotype	Vascular calcification	Reference
			Biogenesis	Mitochondrial dynamics	mitophagy	Metabolic reprogramming	Oxidative stress	Senescence					
Irisin	√	√			√		√			SIRT3	Runx2,Col1,Opn,Ocn	↓	^15^
PGC-1α	√	√	√					√		SIRT3, SOD	Runx2,OPN,Sox9	↓	^16^
Metformin	√		√		√		√			PDK4,AMPK	Runx2,BGP BMP-2		^21^
α-Lipoic acid	√					√	√		Gas6 / Axl / Akt			↓	^30^
Melatonin	√			√			√		AMPK/Drp1	Cas-pase 3	ALP,Runx2	↓	^37^
Melatonin	√			√	√		√		AMPK/OPA1	Cas-pase 3	ALP,Runx2	↓	^38^
Insulin-like growth factor I	√				√			√	Nrf2/Sirt3,p16/p21	PINK1			^97^
Estrogen	√	√			√				SIRT1/LKB1/AMPK/Ulk1	Rab9			^98^
MitoQ	√	√					√		Keap1/Nrf2	Bcl-2,Bax		↓	^99^
Intermedin	√						√		AMPK/ SIRT3	SOD2	Runx2,BMP2	↓	^100^
Ecklonia cava	√		√	√	√		√		PGC-1α/NRF2/SIRT3		Runx2,OCN	↓	^101^

However, CKD-related VC develops from a complex and evolving pathogenesis, and it is not practical to restore or reverse it by targeting a single factor. Some scholars posit that actively addressing the ultimate shared pathway of calcification may be a future direction^102^ In summary, mitochondrial dysfunction has been established as a major cause of vascular calcium deposition. Consequently, this article focuses on the influence of mitochondrial homeostasis, metabolic recoding, ROS, and cellular senescence on the osteogenic phenotype of VSMCs. Nevertheless, further exploration and validation are required to determine the applicability of these novel findings in the clinical treatment of VC.

## Disclosure

All the authors declared no competing interests.
